# Intraoperative Magnesium Sulfate and Early Postoperative Analgesia in Lumbar Microdiscectomy: A Retrospective Clinical Study Integrating Molecular Docking and Protein Interaction Network Analysis

**DOI:** 10.3390/jcm15082888

**Published:** 2026-04-10

**Authors:** Tamer Tamdogan, Ersin Guner, Ilke Tamdogan, Sevim Ondul, Muharrem Furkan Yuzbasi, Ibrahim Yilmaz, Hanefi Ozbek

**Affiliations:** 1 Department of Neurosurgery, Giresun University Faculty of Medicine, Giresun 28200, Türkiye; tamer.tamdogan@giresun.edu.tr (T.T.); sevim.ondul@giresun.edu.tr (S.O.); 2 Department of Pharmacy, Konya Numune Hospital, Ministry of Health of the Republic of Türkiye, Konya 42060, Türkiye; ersin.guner@saglik.gov.tr; 3 Department of Anesthesia and Reanimation, Giresun University Faculty of Medicine, Giresun 28200, Türkiye; ilke.tamdogan@giresun.edu.tr; 4 Department of Neurosurgery, Kahramanmaras Sutcu Imam University Faculty of Medicine, Kahramanmaras 46040, Türkiye; furkanyuzbasi@ksu.edu.tr; 5 Unit of Pharmacovigilance, Doctor Ismail Fehmi Cumalioglu City Hospital, Ministry of Health of the Republic of Türkiye, Tekirdag 59020, Türkiye; 6 Department of Medical Pharmacology, Usak University Faculty of Medicine, Usak 64000, Türkiye; hanefi.ozbek@usak.edu.tr

**Keywords:** lumbar microdiscectomy, magnesium sulfate, postoperative pain, perioperative analgesia, molecular docking, NMDA receptor, opioid receptor, protein–protein interaction

## Abstract

**Background:** Magnesium sulfate (MgSO_4_) has been investigated as an adjuvant in perioperative analgesia because of its antagonistic effects on the N-methyl-D-aspartate receptor (NMDA receptor) and its potential to attenuate central sensitization. However, clinical findings regarding its analgesic efficacy remain inconsistent across surgical procedures. Lumbar microdiscectomy is a common spinal procedure in which effective early postoperative pain control is important for patient comfort and early mobilization. This study aimed to evaluate the effect of intraoperative MgSO_4_ administration on early postoperative analgesia and perioperative outcomes in patients undergoing lumbar microdiscectomy. **Methods:** This retrospective single-center cohort study included thirty-eight patients with American Society of Anesthesiologists (ASA) physical status I–II who underwent elective single-level lumbar microdiscectomy under general anesthesia. Patients were divided into two groups according to intraoperative magnesium administration: a control group receiving standard anesthesia without MgSO_4_ (*n* = 19) and an MgSO_4_ group receiving an intravenous MgSO_4_ bolus of 30 mg/kg followed by a continuous infusion of 10 mg/kg/h until skin closure (*n* = 19). Postoperative pain intensity was assessed using the Numeric Rating Scale (NRS) at 0, 5, 10, 15, and 30 min after admission to the post-anesthesia care unit. Secondary outcomes included intraoperative remifentanil consumption, extubation time, and time to first mobilization. Complementary in silico analyses included molecular docking and protein–protein interaction (PPI) network analysis. **Results:** Postoperative NRS scores were numerically lower in the MgSO_4_ group; however, between-group differences were not statistically significant. Mean intraoperative remifentanil consumption was numerically lower in the MgSO_4_ group (236 ± 166 µg) compared with the control group (319 ± 298 µg), without statistical significance (*p* = 0.27). Repeated-measures analysis demonstrated the significant effect of time on postoperative NRS scores, whereas the overall group effect was not significant. Molecular analyses indicated stable morphine binding to opioid receptors and highlighted glutamatergic signaling components as central nodes within the interaction network. **Conclusions:** Intraoperative MgSO_4_ administration was not associated with significant improvements in early postoperative pain scores or perioperative recovery parameters following lumbar microdiscectomy. Molecular analyses provide exploratory in silico insights and should be interpreted cautiously given the retrospective design and the in silico nature of these findings.

## 1. Introduction

Throughout history, humankind has continuously sought to understand the nature of pain and develop effective strategies for its management [1]. Despite major advances in modern medicine, including the development of novel analgesic agents, refined anesthesia techniques, and improved perioperative monitoring systems, postoperative pain remains a substantial clinical challenge [2,3]. Adequate control of postoperative pain is essential not only for patient comfort but also for optimizing recovery, reducing postoperative complications, and facilitating early mobilization. Nevertheless, even with contemporary multimodal analgesia protocols, a considerable proportion of patients continue to experience moderate to severe pain following surgery.

The persistence of postoperative pain reflects a complex interplay of physiological mechanisms triggered by surgical tissue injury. Surgical trauma initiates a cascade of inflammatory responses characterized by the release of cytokines, prostaglandins, and other inflammatory mediators that activate peripheral nociceptors. This process contributes to peripheral sensitization and promotes enhanced nociceptive transmission to the central nervous system. In addition, central sensitization within the spinal cord and supraspinal structures amplifies pain perception and prolongs nociceptive signaling. The severity of postoperative pain is influenced by multiple factors, including the magnitude of surgical trauma, neuroendocrine stress responses, individual pain thresholds, and interindividual variability in endogenous pain modulation pathways [4]. These multifactorial determinants highlight the need for continued refinement of multimodal analgesic strategies and the exploration of pharmacological adjuvants capable of attenuating central sensitization.

Lumbar disc herniation surgery represents a clinically relevant model for the investigation of postoperative pain and analgesic interventions [5]. Although the primary aim of lumbar microdiscectomy is the decompression of compressed nerve roots and relief of radicular symptoms, the surgical approach inevitably involves manipulation of paraspinal muscles, fascia, and osseous structures within the lumbar spine. This local tissue trauma may induce inflammatory responses, edema, and nociceptive signaling that contribute to postoperative discomfort. Furthermore, the lumbar spine plays a central biomechanical role in weight-bearing and movement, which may exacerbate postoperative pain during the early recovery period. Inadequate pain control in this patient population may delay mobilization, increase opioid requirements, and potentially prolong functional recovery.

In response to these challenges, a variety of perioperative strategies have been proposed to improve pain management in lumbar spine surgery. These approaches include multimodal analgesia protocols, regional anesthesia techniques, non-opioid pharmacological adjuvants, and optimization of intraoperative anesthetic regimens [6,7]. The primary objectives of these strategies are to reduce postoperative pain intensity, limit opioid consumption and opioid-related adverse effects, and enhance early postoperative recovery.

Among pharmacological adjuvants, magnesium sulfate (MgSO_4_) has attracted considerable interest in recent years as a potential component of multimodal analgesia [8,9]. Magnesium exerts several pharmacological effects relevant to pain modulation, most notably through antagonism of the N-methyl-D-aspartate (NMDA) receptor and inhibition of calcium influx into neuronal cells. By limiting NMDA receptor-mediated excitatory neurotransmission, MgSO_4_ may attenuate central sensitization and reduce the amplification of nociceptive signaling. In addition to these effects on glutamatergic pathways, magnesium has been shown to modulate presynaptic neurotransmitter release and influence opioid receptor signaling pathways, mechanisms that may contribute to enhanced analgesic efficacy in combination with opioid analgesics. Several randomized controlled trials and meta-analyses have suggested that perioperative intravenous MgSO_4_ administration may reduce postoperative opioid consumption and improve early postoperative pain scores across surgical populations [10,11,12]. Recent meta-analyses and contemporary clinical studies have reported heterogeneous findings regarding perioperative magnesium administration; some report modest reductions in postoperative pain and opioid consumption, whereas others emphasize the need for standardized dosing strategies and further high-quality trials, particularly in spinal surgery populations [10,11,12].

Despite these promising observations, concerns remain regarding the perioperative use of MgSO_4_ during general anesthesia. Magnesium is known to potentiate the effects of non-depolarizing neuromuscular blocking agents and may prolong the duration of neuromuscular blockade, particularly when agents such as rocuronium are used [13,14]. Experimental studies have also suggested that magnesium administration may influence the pharmacodynamic interaction between neuromuscular blockers and reversal agents such as sugammadex [15]. These potential interactions highlight the importance of carefully evaluating both the analgesic benefits and the safety profile of perioperative MgSO_4_ administration.

Another factor contributing to the heterogeneity of findings in the literature is methodological variability among clinical studies. Differences in patient populations, surgical procedures, anesthetic protocols, magnesium dosing regimens, timing of administration, and outcome measurements may substantially influence study results. Furthermore, the absence of standardized neuromuscular monitoring in some clinical investigations complicates the interpretation of safety outcomes. Although the analgesic effects of MgSO_4_ have been investigated in several surgical contexts, evidence specifically addressing lumbar disk surgery remains limited.

Consequently, the clinical effectiveness and perioperative implications of MgSO_4_ administration in patients undergoing lumbar microdiscectomy remain uncertain. Clarifying this issue is particularly relevant given the high prevalence of lumbar spine procedures and the potential benefits of improved postoperative pain control on early recovery and functional outcomes.

To provide exploratory systems-level context for the clinical findings, advances in systems biology and network pharmacology have provided new opportunities to investigate complex biological interactions underlying nociceptive processing and analgesic modulation. In particular, protein–protein interaction (PPI) network analysis has emerged as a valuable approach for identifying functional relationships among genes and proteins involved in neuronal signaling pathways. Functional enrichment analysis based on Gene Ontology (GO) terms enables identification biological processes (BP) and molecular functions associated with specific protein networks, thereby providing a broader systems-level perspective on potential pharmacological mechanisms. Furthermore, topological network analysis can identify highly connected “hub” proteins that may play central regulatory roles within these interaction networks. Among the available algorithms, maximal clique centrality (MCC) has been shown to be particularly effective for detecting biologically relevant hub proteins within complex interactomes. In the context of pain modulation, such approaches may help elucidate how glutamatergic signaling pathways, intracellular kinase cascades, and neurotrophic signaling networks interact with opioid receptor-mediated analgesic mechanisms. Therefore, integrating molecular docking findings with PPI network analysis, GO functional enrichment, and MCC-based hub protein identification may provide additional mechanistic insight into the molecular pathways potentially involved in magnesium-mediated modulation of nociceptive signaling. However, clinical findings regarding the analgesic efficacy of perioperative magnesium remain inconsistent [10,11].

Accordingly, the present study aimed to evaluate whether intraoperative MgSO_4_ administration, in addition to standard morphine-based analgesia, provides clinically meaningful benefits in patients undergoing lumbar microdiscectomy.

The effects on early postoperative pain scores, intraoperative opioid consumption, extubation time, and time to first mobilization were evaluated. In addition, complementary in silico analyses including molecular docking and PPI network analysis with functional enrichment were performed to provide exploratory mechanistic context for magnesium-mediated modulation of nociceptive signaling.

## 2. Materials and Methods

### 2.1. Study Design

This study was designed as a single-center retrospective cohort analysis based on archived perioperative anesthesia and postoperative records of patients who underwent elective single-level lumbar microdiscectomy. Due to the retrospective design, no prospective randomization, allocation concealment, or blinding procedures were implemented. To minimize analytical bias, all patient records were anonymized and numerically coded prior to statistical analysis.

Investigators responsible for statistical evaluation were blinded to intraoperative magnesium sulfate (MgSO_4_) administration status during data analysis. The study was conducted and reported in accordance with the Strengthening the Reporting of Observational Studies in Epidemiology (STROBE) Statement [16], and the STROBE checklist was followed during manuscript preparation. Given the retrospective design and the relatively small sample size, the results should be interpreted with caution and may not be generalizable to broader surgical populations. Magnesium administration was not based on a predefined institutional protocol; instead, it reflected routine clinical practice and was determined by the attending anesthesiologist according to individual clinical judgment regarding anticipated postoperative pain and multimodal analgesia preference. Consequently, group allocation was non-random and may be subject to selection bias, which was explored using expanded baseline comparability assessment and sensitivity analyses.

### 2.2. Study Population

Medical records of 76 adult patients aged between 18 and 70 years who underwent elective single-level lumbar microdiscectomy under general anesthesia between March 2025 and September 2025 were retrospectively reviewed. Eligible patients were classified as displaying American Society of Anesthesiologists (ASA) physical status I–II.

The exclusion criteria were defined to minimize potential confounding factors that could influence neuromuscular transmission, anesthetic pharmacodynamics, or postoperative pain outcomes. Patients were excluded if they had severe renal insufficiency (creatinine clearance < 30 mL/min), neuromuscular disorders including myasthenia gravis, chronic opioid use for longer than three months, pregnancy, or a requirement for repeated spinal surgery. Additional exclusion criteria included severe atrioventricular block or uncontrolled cardiac arrhythmia, the use of medications known to affect neuromuscular transmission or anesthetic pharmacodynamics (such as calcium channel blockers, hypnotics, anxiolytics, or antipsychotics), and any known hypersensitivity or contraindication to morphine or MgSO_4_. Additional baseline and perioperative variables were extracted from medical records, including preoperative pain scores (NRS), symptom duration, operated disc level (L4–L5 or L5–S1), surgery duration, ASA status, comorbidities, and preoperative analgesic use.

**Figure 1:**
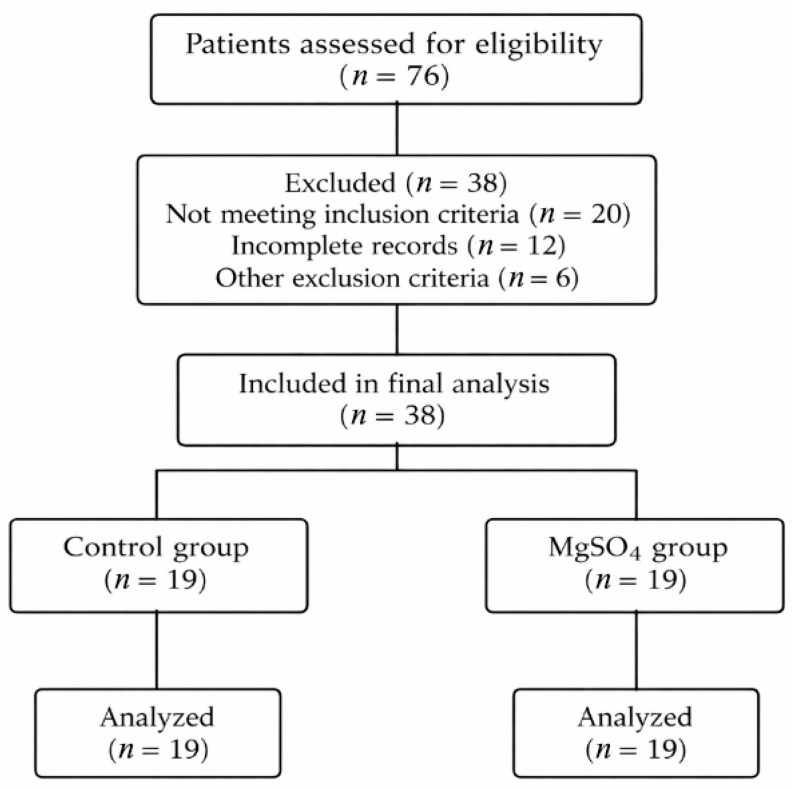
Flow diagram of patient selection and group allocation.

After applying the exclusion criteria, 38 patients were included in the final analysis and categorized into two groups according to intraoperative magnesium administration (Figure 1): a Control group receiving standard anesthesia without MgSO_4_ (*n* = 19) and an MgSO_4_ group receiving intraoperative MgSO_4_ infusion (*n* = 19). All procedures included were performed by the same experienced neurosurgeon. This resulted from the retrospective selection of eligible cases and may have reduced inter-operator variability.

Preoperative analgesic use was uniform across the cohort, with all patients receiving nonsteroidal anti-inflammatory drugs (NSAIDs) preoperatively. Group allocation was therefore non-random and reflected routine clinical decision-making.

A total of 76 patients undergoing elective single-level lumbar microdiscectomy were screened. After applying exclusion criteria, 38 patients were included in the final analysis and allocated to the Control group (*n* = 19) or the MgSO_4_ group (*n* = 19).

Given the small sample size, the study may have been underpowered to detect small but clinically relevant between-group differences. Consequently, smaller but potentially clinically relevant differences between groups may not have been detectable within the present cohort.

### 2.3. Anesthetic Management

All patients received intravenous midazolam (0.03 mg/kg) as premedication. General anesthesia was induced with propofol (2 mg/kg), fentanyl (2 µg/kg), and rocuronium (0.6 mg/kg). Anesthesia was maintained with sevoflurane at approximately one minimum alveolar concentration (MAC), combined with a continuous remifentanil infusion (0.10–0.25 µg/kg/min).

Patients in the MgSO_4_ group received intraoperative MgSO_4_ administration consisting of an intravenous bolus of 30 mg/kg administered over 15 min following anesthetic induction, followed by a continuous infusion of 10 mg/kg/h maintained until surgical skin closure.

All patients received standardized multimodal analgesia including intravenous morphine (0.05 mg/kg) and paracetamol (1 g) approximately 30 min before the end of surgery. Neuromuscular blockade was reversed with sugammadex (2 mg/kg), and tracheal extubation was performed after confirming adequate neuromuscular recovery using standard clinical criteria, including sustained head lift, adequate handgrip strength, spontaneous tidal breathing, and peripheral oxygen saturation (SpO_2_) greater than 95%.

Total intraoperative remifentanil consumption and extubation time were recorded for each patient. Perioperative anesthesia records were also reviewed for MgSO_4_-related adverse events, including arrhythmia, clinically suspected delayed neuromuscular recovery, or postoperative respiratory complications.

### 2.4. Outcome Measures

The primary outcomes of the study were postoperative pain intensity and total intraoperative remifentanil consumption. Postoperative pain was assessed using the Numeric Rating Scale (NRS), which ranges from 0 (no pain) to 10 (worst imaginable pain). NRS scores were recorded at 0, 5, 10, 15, and 30 min after arrival in the post-anesthesia care unit (PACU) [17]. The 0-min time point was defined as the first pain assessment performed immediately upon admission to the PACU following tracheal extubation.

Secondary outcomes included extubation time, defined as the interval between cessation of anesthetic administration and successful tracheal extubation, and time to first mobilization, defined as the interval between PACU admission and the first documented assisted ambulation. In addition, perioperative records were reviewed to identify MgSO_4_-related adverse events, including hemodynamic instability, cardiac arrhythmia, clinically suspected delayed neuromuscular recovery, or postoperative respiratory complications. The analysis focused specifically on the early postoperative recovery period in the PACU, during which pain assessments were systematically recorded at predefined time points. Analgesic consumption beyond intraoperative remifentanil use was not included in the predefined early PACU outcome assessment.

### 2.5. Computerized Molecular Analysis

Complementary in silico analyses were performed to provide exploratory mechanistic context for magnesium-mediated modulation of nociceptive signaling. These analyses included molecular docking simulations and PPI network analysis. Molecular docking simulations were conducted to evaluate the binding interactions of morphine with μ-, δ-, and κ-opioid receptors using AutoDock 4.2.6, and the resulting binding conformations were visualized and analyzed using MGLTools 1.5.6. In addition, structural features of the NMDA receptor channel were examined to investigate the potential positioning of Mg^2+^ ions within the receptor pore. Detailed descriptions of the docking procedures and structural analyses are provided in the following subsections.

#### 2.5.1. Molecular Docking Analyses

Molecular docking analyses were performed to evaluate the interactions of morphine with the μ-, δ-, and κ-opioid receptors, which play central roles in analgesic signaling pathways. The three-dimensional structures of these receptors were obtained from the Protein Data Bank (PDB), including the μ-opioid receptor (PDB ID: 8EF6) [18], the δ-opioid receptor (PDB ID: 6PT2) [19], and the κ-opioid receptor (PDB ID: 6VI4) [20].

Docking simulations were performed for morphine interactions with opioid receptors using AutoDock 4.2.6, and the resulting binding conformations were visualized and analyzed using MGLTools 1.5.6. For each ligand–receptor system, twenty independent docking runs were generated and ranked according to predicted binding energy, with the lowest-energy conformations selected for structural analysis.

##### Morphine–Opioid Receptor Docking

Docking simulations were performed to evaluate the binding interactions of morphine with the μ-, δ-, and κ-opioid receptors. The receptor structures used in the simulations were the μ-opioid receptor (PDB ID: 8EF6), the δ-opioid receptor (PDB ID: 6PT2), and the κ-opioid receptor (PDB ID: 6VI4) [18,19,20]. The ligand structure of morphine was prepared using ChemBio Ultra 13.0, and energy minimization was performed using the MM2 force field prior to docking simulations.

##### NMDA Receptor Mg^2+^ Blockade Analysis

To explore structural aspects related to magnesium interactions within nociceptive signaling pathways, an additional analysis was performed focusing on the human GluN1/GluN2B NMDA receptor structure (PDB ID: 9IYP) [21]. This analysis aimed to evaluate the potential positioning of Mg^2+^ ions within the receptor channel pore under resting membrane potential conditions, consistent with the established voltage-dependent magnesium blockade mechanism of NMDA receptors.

Detailed descriptions of receptor and ligand structure preparation, docking grid parameters, redocking validation procedures, and additional molecular docking results are provided in the Supplementary Materials (Supplementary File S1).

#### 2.5.2. Construction of the PPI Network

To investigate potential molecular interactions among analgesia-related targets, a PPI network was constructed using the STRING database (Search Tool for the Retrieval of Interacting Genes/Proteins; version 12.0; https://string-db.org) (accessed on 7 April 2026) [22].

STRING integrates experimentally validated and predicted protein interactions derived from experimental data, computational prediction methods, curated biological databases, gene co-expression analysis, and automated text mining of scientific literature.

Target proteins were selected based on literature evidence linking opioid receptor signaling, glutamatergic neurotransmission, calcium channel activity, and intracellular kinase pathways to nociceptive processing and synaptic plasticity. The following proteins were included in the analysis based on their reported involvement in nociceptive transmission and neuronal signaling pathways: OPRM1, OPRD1, GRIN1, GRIN2B, GRIA1, GRIA2, CACNA1A, CACNA1B, CAMK2A, MAPK1, AKT1, CREB1, and BDNF. The organism was restricted to Homo sapiens. The interaction network was generated using the multiple-protein query option with a minimum interaction score of 0.400 (medium confidence). A medium confidence score (0.400) was selected to capture biologically relevant functional interactions while avoiding excessive network sparsity that may occur with higher confidence thresholds.

Interaction evidence was derived from multiple STRING evidence channels including experimentally determined interactions, curated biological databases, gene co-expression data, and literature-based text mining. No additional interacting proteins were added in order to preserve the predefined protein set and avoid potential network expansion bias. Restricting the analysis to a predefined protein set ensured that the resulting network primarily reflected established analgesia-related signaling pathways rather than secondary interactors that may not be directly involved in nociceptive modulation.

The PPI enrichment *p*-value was automatically calculated by the STRING database to evaluate whether the observed number of interactions among the selected proteins was greater than expected for a random protein set of similar size.

Functional enrichment analysis was performed within the STRING platform using Gene Ontology (GO), Kyoto Encyclopedia of Genes and Genomes (KEGG), and Reactome pathway databases. Enrichment significance was calculated using the STRING statistical framework with false discovery rate (FDR) correction, and enriched terms with FDR < 0.05 were considered statistically significant. The generated PPI network was exported from STRING and subsequently imported into Cytoscape software (version 13.4) for visualization and further topological network analysis [23].

#### 2.5.3. Network Visualization and Topological Analysis

Network visualization and quantitative topological analysis were performed using Cytoscape for analysis of biological interaction networks [23].

Topological properties of the network were calculated using the NetworkAnalyzer tool implemented in Cytoscape. To assess the topological importance of individual proteins within the interaction network, several centrality measures were examined, including degree centrality, betweenness centrality, and closeness centrality. Degree centrality reflects the number of direct interactions associated with each node and provides an estimate of the immediate connectivity of a given protein. Betweenness centrality indicates the extent to which a protein functions as a bridge connecting different regions of the network and may therefore influence information flow within the interaction structure. Closeness centrality, in contrast, reflects how close a node is to all other nodes within the network topology and provides an indication of its overall accessibility within the system. Together, these complementary metrics were used to characterize the structural relevance and connectivity patterns of proteins within the PPI network.

#### 2.5.4. Identification of Pivotal Proteins

Hub proteins within the interaction network were identified using the cytoHubba plugin in Cytoscape [24].

In the present study, hub proteins were ranked using the MCC algorithm, which has been demonstrated to be effective in identifying essential proteins within complex interaction networks. Proteins with the highest MCC scores were considered hub proteins, reflecting their central topological roles within the interaction network.

### 2.6. Statistical Analysis

All statistical analyses were performed using validated statistical software. Continuous variables were summarized as mean ± standard deviation (SD) or median (interquartile range, IQR) as appropriate. Categorical variables were presented as counts and percentages.

Normality of distributions was assessed using the Shapiro–Wilk test, and homogeneity of variances was evaluated using Levene’s test. Between-group baseline comparability was assessed not only using *p*-values but also standardized mean differences (SMD), with an absolute SMD < 0.1 considered indicative of negligible imbalance.

The primary clinical analysis evaluated the trajectory of postoperative pain scores during the early post-anesthesia care unit (PACU) period. This was performed using a linear mixed-effects model with group (MgSO_4_ vs. control), time (0, 5, 10, 15, and 30 min), and group × time interaction as fixed effects, and subject as a random intercept to account for within-patient correlation.

When the interaction term was explored, pairwise contrasts between groups at each time point were evaluated using Holm-corrected post hoc comparisons to control for multiple testing. Estimated marginal means and mean differences with 95% confidence intervals (CI) were reported.

As a secondary summary measure of early postoperative pain burden, the area under the curve (AUC) of NRS scores during the first 30 min in the PACU was calculated for each patient using the trapezoidal method. Between-group comparisons for AUC were performed using independent-samples tests and reported as mean differences with 95% CI.

Continuous perioperative outcomes including intraoperative remifentanil consumption, extubation time, and time to first mobilization were compared between groups using independent-samples *t*-tests or Mann–Whitney U tests as appropriate. Results were expressed as mean differences with 95% confidence intervals and Cohen’s d effect sizes.

To address potential confounding inherent to the retrospective design, exploratory multivariable linear regression models were constructed as sensitivity analyses adjusting for baseline covariates available in the archived records, including age, sex, body mass index, preoperative pain score, symptom duration, operated disk level, surgery duration, ASA status, comorbidities, and preoperative analgesic use.

These analyses were prespecified as sensitivity analyses and not intended for causal inference.

Two-sided *p*-values < 0.05 were considered statistically significant.

## 3. Results

### 3.1. Patient Demographics and Baseline Characteristics

Baseline comparability was assessed using both *p*-values and standardized mean differences (SMD). Baseline characteristics, including demographic and perioperative variables, were comparable between groups. SMD for all variables were below conventional imbalance thresholds, indicating adequate baseline comparability (Table 1).

### 3.2. Early Postoperative Pain Trajectory (NRS)

**Figure 2:**
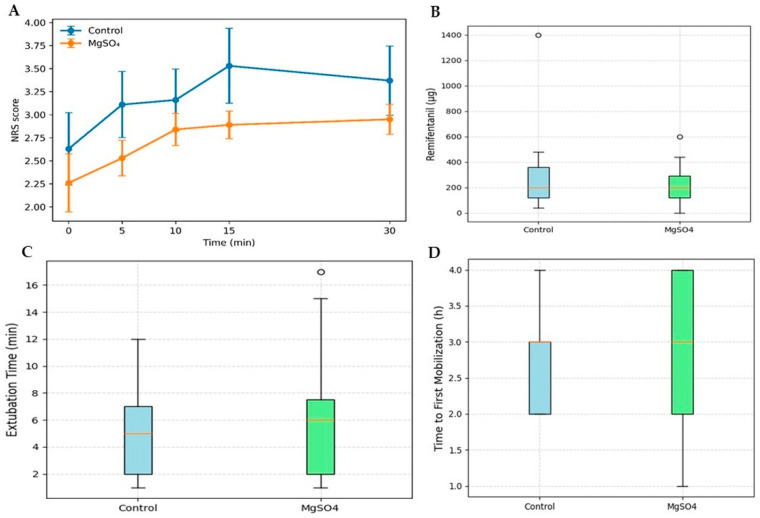
(**A**) Early postoperative NRS trajectory in the Control and MgSO_4_ groups during the first 30 min after PACU admission. Data are presented as mean ± SEM. (**B**) Intraoperative remifentanil consumption in both groups. The mean difference was −83 µg (95% CI −264.09 to 29.00). (**C**) Extubation time following surgery. The mean difference between groups was 1.37 min (95% CI −1.53 to 4.43). (**D**) Time to first mobilization. The mean difference was 0.06 h (95% CI −0.50 to 0.70). Circles represent outliers, and the horizontal lines within boxes indicate median values.

A linear mixed-effects model was used to evaluate the trajectory of postoperative NRS scores during the early PACU period. The model included group, time, and group × time interaction as fixed effects and subject as a random effect. A significant effect of time on NRS scores was observed, indicating decreasing pain intensity over time. However, no significant main effect of group was detected (Figure 2A).

The group × time interaction did not remain statistically significant after model estimation. Holm-corrected post hoc comparisons between groups at each time point showed no statistically significant differences. Mean differences between groups at individual time points were small, and all corresponding 95% confidence intervals crossed zero.

These findings suggest that although pain scores were numerically lower in the MgSO_4_ group at several time points, the magnitude of these differences was modest and imprecisely estimated.

### 3.3. Intraoperative Remifentanil Consumption

Mean intraoperative remifentanil consumption was numerically lower in the MgSO_4_ group. The mean difference was −83 µg (95% CI −264.09 to 29.00), indicating no statistically significant reduction. Although the point estimate favored magnesium, the confidence interval was wide and crossed zero (Figure 2B).

### 3.4. Extubation Time and Time to First Mobilization

Extubation time was slightly longer in the MgSO_4_ group. The mean difference was 1.37 min (95% CI −1.53 to 4.43), indicating no statistically significant difference (Figure 2C).

Time to first mobilization was similar between groups. The mean difference was 0.06 h (95% CI −0.50 to 0.70), with no statistically significant difference (Figure 2D, Table 2).

### 3.5. Safety Outcomes

No clinically apparent MgSO_4_-related adverse events were documented in the anesthesia records during the intraoperative or early postoperative period. Because objective neuromuscular monitoring was not routinely reported, subtle prolongation of neuromuscular blockade cannot be excluded. No clinically meaningful differences were observed between groups in extubation time or time to first mobilization. These findings should be interpreted cautiously given the retrospective design and limited sample size.

Additional propensity score and IPTW-based sensitivity analyses yielded similar findings and are provided in the Supplementary Materials.

### 3.6. Computerized Molecular Analysis

#### 3.6.1. Molecular Docking

##### Morphine–Opioid Receptor Interactions

Molecular docking simulations suggested that morphine may interact with μ-, δ-, and κ-opioid receptors, with predicted binding energies ranging from −8.75 to −10.38 kcal/mol. The strongest predicted binding affinity was observed for the κ-opioid receptor (PDB ID: 6VI4) (Table 3). Several potential hydrogen-bond interactions between morphine and amino acid residues within receptor binding pockets were identified. The calculated RMSD values ranged from 0.06–0.08 Å, supporting internal docking consistency. These in silico findings should be interpreted as hypothesis-generating and do not establish in vivo pharmacological effects.

Docking results are presented for structural interpretation and should not be considered quantitative predictors of clinical analgesic efficacy.

In the μ-opioid receptor structure (PDB ID: 8EF6), predicted hydrogen-bond interactions involved Tyr328 and Gln126 residues. For the δ-opioid receptor (PDB ID: 6PT2), potential hydrogen bonds included Tyr308 and Asp128 residues. In the κ-opioid receptor structure (PDB ID: 6VI4), morphine was predicted to interact through hydrogen bonds with Ser323 and Thr111 residues.

**Figure 3:**
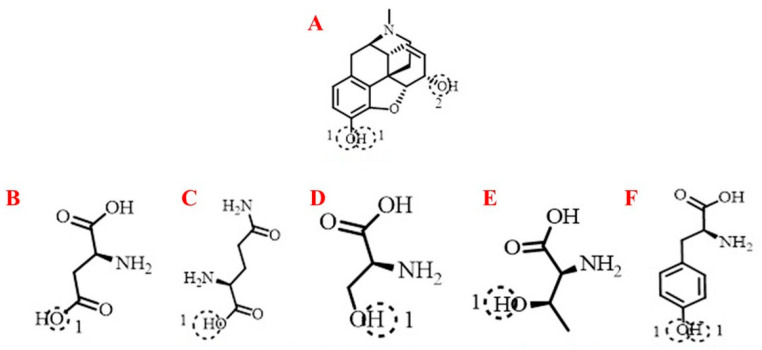
Representation of morphine binding interactions with selected amino acid residues at μ, δ, and κ opioid receptors. (**A**) Morphine; (**B**) Aspartic acid; (**C**) Glutamine (GLN); (**D**) Serine (SER); (**E**) Threonine (THR); (**F**) Tyrosine (TYR). These schematic representations illustrate potential interactions within receptor binding pockets (Figure 3 and Figure 4). Dashed circles indicate potential hydrogen-bond interaction regions.

**Figure 4:**
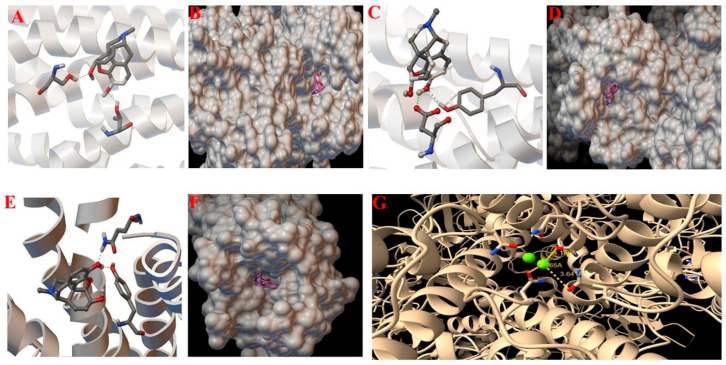
(**A**–**F**) Representative docking poses of morphine within opioid receptor binding pockets generated using MGLTools 1.5.6. (**G**) Structural visualization of Mg^2+^ positioning within the NMDA receptor structure (PDB ID: 9IYP).

Two-dimensional (2D) and three-dimensional (3D) representations of the docking poses illustrate potential interactions within the receptor binding pockets, including Tyr, Ser, Thr, Gln, and Asp residues (Figure 3 and Figure 4).

##### NMDA Receptor Mg^2+^ Blockade

Structural analysis based on the human GluN1/GluN2B NMDA receptor model (PDB ID: 9IYP) suggested that Mg^2+^ ions may occupy the receptor channel pore under resting membrane potential conditions. The Mg^2+^ ion was predicted to form coordination interactions with the carbonyl oxygen atoms of Asn616 (GluN1) and Asn615 (GluN2B), as well as with Asp136 and Asp181 residues. The observed Mg^2+^–O interaction distances ranged from 2.77 to 4.19 Å, consistent with octahedral coordination geometry. Structural representations of morphine-receptor docking complexes are shown in Figure 3 and Figure 4A–F, whereas the Mg^2+^ positioning within the NMDA receptor channel is illustrated in Figure 4G. Protein surfaces are shown in beige, ligands in gray, and interacting residues in colored sticks. Mg^2+^ is shown as a green sphere.

#### 3.6.2. PPI Network Analysis

**Figure 5:**
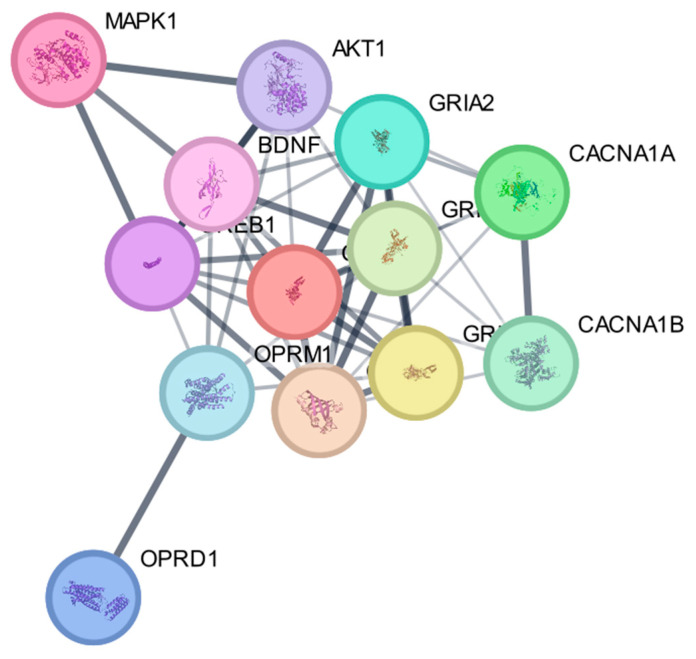
PPI network of analgesia-related targets generated using the STRING database (version 12.0) for Homo sapiens. Nodes represent proteins and edges indicate predicted functional associations. Edge thickness corresponds to interaction confidence.

A PPI network of the selected nociception-related proteins was generated using the STRING database (Figure 5). The resulting network consisted of 13 nodes and 44 edges, suggesting a relatively interconnected network structure among the analyzed proteins.

The network exhibited an average node degree of 6.77 and an average local clustering coefficient of 0.782, suggesting a high degree of connectivity and clustering within the interaction network. For a random protein set of comparable size, the expected number of edges was 8, whereas the observed network contained 44 interactions, resulting in a highly significant PPI enrichment *p*-value < 1.0 × 10^−16^. This result suggests that the analyzed proteins interact more frequently than expected by chance and may participate in shared biological pathways.

Topological inspection suggested a connected interaction core composed primarily of proteins involved in glutamatergic neurotransmission and intracellular signaling pathways, including GRIN1, GRIN2B, GRIA1, GRIA2, CAMK2A, CREB1, and BDNF (Figure 5). These proteins showed multiple interactions with intracellular signaling regulators such as AKT1 and MAPK1. In contrast, the opioid receptors OPRM1 and OPRD1 appeared more peripherally located within the network topology.

This topological arrangement suggests that glutamatergic receptors and downstream intracellular signaling components may form a central interaction module, whereas opioid receptors occupy a more peripheral position.

#### 3.6.3. Identification of Pivotal Proteins

**Figure 6:**
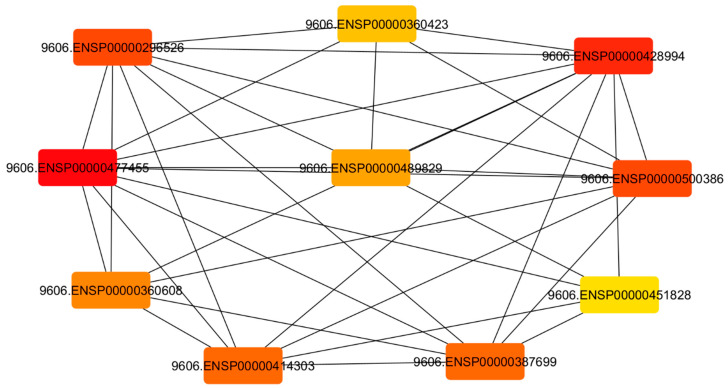
Identification of hub proteins in the PPI network using cytoHubba MCC analysis in Cytoscape. Hub proteins were prioritized using the MCC algorithm implemented in the cytoHubba plugin. Nodes are colored according to their MCC scores, with warmer colors indicating higher-ranking hub proteins.

Topological analysis of the PPI network was performed using Cytoscape NetworkAnalyzer together with the cytoHubba plugin to identify potentially highly connected nodes within the interaction network (Figure 6).

Among the analyzed proteins, GRIN2B exhibited the highest degree value (10), suggesting that it may interact with a large number of proteins within the network (Table 4).

Other highly connected proteins included *GRIA1*, *CREB1*, and *BDNF* (degree = 9), followed by *CAMK2A* (degree = 8) and *GRIN1* and *GRIA2* (degree = 7). These proteins also displayed relatively high closeness centrality values, reflecting their central positions within the interaction network.

In contrast, OPRM1 and OPRD1 exhibited lower degree values (3 and 1, respectively), suggesting a more peripheral topological position. Overall, these findings suggest that glutamate receptors (GRIN and GRIA family proteins), synaptic signaling regulators (CAMK2A and CREB1), and neurotrophic signaling components (BDNF) may contribute to the central interaction module of the network.

#### 3.6.4. Functional Enrichment Analysis

Functional enrichment analysis suggested that the identified protein network was associated with BP related to synaptic signaling and neuronal communication (Table 5).

Among Gene Ontology BP, the most enriched terms included chemical synaptic transmission (GO:0007268; FDR = 8.28 × 10^−9^), modulation of chemical synaptic transmission (FDR = 1.63 × 10^−7^), and regulation of postsynaptic membrane potential (FDR = 7.34 × 10^−6^). These findings suggest that the analyzed proteins may be involved in neuronal synaptic activity.

Molecular function enrichment analysis identified ionotropic glutamate receptor activity and calcium channel activity as enriched categories, suggesting potential involvement of glutamatergic neurotransmission and calcium signaling within the network.

KEGG pathway analysis suggested enrichment in neuronal signaling pathways, including dopaminergic synapse, long-term potentiation, nicotine addiction, and amphetamine addiction.

Reactome pathway analysis indicated enrichment in post-NMDA receptor activation events and CREB1 phosphorylation through NMDA receptor-mediated signaling, suggesting potential involvement of glutamatergic signaling cascades and downstream transcriptional regulation. Collectively, these results suggest that the identified protein interaction network may be associated with synaptic plasticity, calcium-dependent signaling pathways, and neurotransmitter-mediated neuronal communication, which are relevant to nociceptive processing and analgesic modulation.

Taken together, the molecular docking analyses and network-based systems biology approach suggest that the analyzed protein set forms an interconnected interaction network centered on glutamatergic signaling components and downstream intracellular signaling regulators. The identification of GRIN2B, CREB1, BDNF, and CAMK2A as central nodes, together with enrichment of synaptic signaling and calcium-dependent pathways, is consistent with a coordinated molecular framework potentially related to nociceptive transmission and synaptic plasticity.

## 4. Discussion

In this single-center retrospective cohort study, intraoperative MgSO_4_ infusion did not result in statistically significant reductions in early postoperative pain scores, intraoperative remifentanil consumption, extubation time, or time to first mobilization in patients undergoing elective single-level lumbar microdiscectomy. Nevertheless, modest trends toward lower postoperative NRS scores and reduced intraoperative opioid requirements were observed in the MgSO_4_ group; however, these differences did not reach statistical significance and should be interpreted cautiously.

Postoperative pain trajectories were evaluated using a linear mixed-effects model. The group × time interaction did not remain statistically significant after model estimation, and Holm-corrected post hoc comparisons at individual time points showed no statistically significant between-group differences. A gradual increase in NRS scores during the early PACU period was observed in both groups. This pattern may reflect the progressive resolution of intraoperative anesthetic and opioid effects rather than a true worsening of postoperative pain. During the immediate post-anesthesia phase, residual analgesic and sedative effects can transiently suppress pain perception, and pain scores may increase as these effects diminish and patients regain full consciousness. Similar early postoperative pain trajectories have been described in studies evaluating analgesic interventions in short surgical procedures.

Magnesium is known to exert analgesic effects primarily through functional NMDA receptor antagonism and modulation of presynaptic calcium influx, thereby attenuating excitatory neurotransmission and reducing acetylcholine release at the neuromuscular junction [12,25]. Clinical studies across various surgical models, including spine, abdominal, and gynecologic procedures, have reported reductions in postoperative pain scores and opioid consumption following perioperative MgSO_4_ administration [26,27,28,29]. However, the overall evidence remains heterogeneous. Meta-analyses suggest that the magnitude of the analgesic benefit is strongly influenced by factors such as surgical nociceptive burden, MgSO_4_ dosing strategy, intraoperative opioid regimens, and the background multimodal analgesia protocol [10,30].

The present findings are broadly consistent with these observations. Single-level lumbar microdiscectomy represents a relatively low-nociceptive surgical procedure, in which postoperative pain is predominantly associated with localized tissue manipulation rather than extensive NMDA receptor-mediated central sensitization. This pathophysiological context may partly explain why intraoperative MgSO_4_ administration did not produce measurable improvements in early postoperative clinical outcomes despite established pharmacological plausibility at the receptor level.

The complementary molecular analyses performed in the present study provide exploratory systems-level context for interpreting these clinical findings. Morphine docking was specifically performed because perioperative morphine constituted the primary opioid analgesic administered in the clinical cohort, allowing the computational analyses to reflect the pharmacological conditions of the clinical protocol. Structural modeling of the human GluN1/GluN2B NMDA receptor supported stable positioning of Mg^2+^ within the receptor channel pore under resting membrane potential conditions, which is consistent with the well-established voltage-dependent magnesium blockade mechanism of NMDA receptors.

These findings support the structural plausibility of morphine–opioid receptor interactions at the molecular level. For NMDA receptor channel activation, both ligand binding and voltage-dependent Mg^2+^ unblocking is required. From a pharmacological perspective, this model is highly relevant in the context of opioid analgesia and tolerance development. Chronic morphine exposure may trigger calcium-dependent intracellular signaling cascades through sustained NMDA receptor activation, a process that has been implicated in opioid tolerance and opioid-induced hyperalgesia. Mg^2+^ blockade may attenuate this mechanism by limiting Ca^2+^ influx through the receptor channel. The NMDA receptor structural model is consistent with the pharmacological plausibility of interactions between NMDA receptor blockade and opioid-mediated analgesia. However, these structural observations did not translate into measurable clinical effects in the present cohort.

The network-based systems biology analyses further supported the central role of glutamatergic signaling pathways in nociceptive modulation. The PPI network showed a highly interconnected structure enriched for synaptic signaling and neuronal communication. Notably, GRIN2B emerged as the most highly connected node within the interaction network, while CREB1 and BDNF were also identified as central hub proteins. This pattern is biologically coherent, as GRIN2B-containing NMDA receptors are closely associated with calcium influx and synaptic plasticity, whereas CREB1 and BDNF represent key downstream mediators of activity-dependent transcriptional and neurotrophic signaling. Together, these findings suggest a potential interaction framework linking NMDA receptor activity to intracellular signaling pathways involved in nociceptive processing and synaptic plasticity.

Functional enrichment analysis further reinforced this interpretation. Enrichment of BP related to chemical synaptic transmission, postsynaptic membrane regulation, ionotropic glutamate receptor activity, and calcium channel activity indicates that the analyzed network is centered on synaptic plasticity and calcium-dependent neuronal signaling. Reactome pathway enrichment involving post-NMDA receptor activation events and CREB1 phosphorylation is consistent with a possible role of glutamatergic signaling in regulating downstream transcriptional responses associated with neuronal adaptation and pain modulation. Although the present study cannot establish a causal relationship between the molecular observations and the clinical outcomes, the integrative analysis provides an exploratory systems-level context that may help explain how NMDA receptor–related signaling pathways interact with opioid-mediated analgesia in the perioperative setting.

Given the relatively small sample size, the study may have been underpowered to detect subtle but clinically meaningful between-group differences. The modest numerical reductions in pain scores and opioid consumption observed in the MgSO_4_ group should therefore be interpreted cautiously and may reflect pharmacological effects that remain below the threshold of statistical detection in this relatively low-intensity surgical model [11,31,32].

Taken together, the present findings suggest that although MgSO_4_ remains pharmacologically plausible as an adjunctive analgesic agent, its clinical impact in minimally invasive lumbar spine procedures appears limited. In routine single-level microdiscectomy, the marginal analgesic contribution of MgSO_4_ should be considered alongside practical factors such as drug preparation, infusion management, and perioperative workflow. MgSO_4_ may therefore be more appropriately reserved for procedures associated with greater nociceptive input or for patient populations at increased risk of opioid tolerance or central sensitization.

Future investigations may help clarify the clinical role of MgSO_4_ in perioperative analgesia. Larger multicenter studies would improve statistical power and generalizability, while extended postoperative follow-up beyond the early PACU period may capture delayed analgesic effects. Integration of perioperative serum Mg^2+^ measurements could help establish pharmacokinetic–pharmacodynamic correlations, and studies involving procedures with higher nociceptive burden, such as multilevel spinal fusion, may better define potential dose–response relationships. Furthermore, combining computational modeling approaches with pharmacokinetic–pharmacodynamic analyses may provide a more comprehensive framework linking molecular mechanisms with systemic analgesic outcomes.

Several limitations of the present study should be acknowledged. First, the retrospective design introduces an inherent susceptibility to selection and information bias and relies on the completeness and accuracy of archived clinical records. Second, the relatively small sample size (*n* = 38) reduces the ability to detect subtle analgesic effects, particularly for variables with substantial interindividual variability such as pain scores and intraoperative opioid consumption. Third, the study population consisted exclusively of patients undergoing single-level lumbar microdiscectomy, which limits extrapolation of the findings to more extensive spinal procedures associated with greater nociceptive stimulation. Fourth, the standardized multimodal analgesic regimen, including perioperative morphine and paracetamol administration, may have attenuated potential independent effects of MgSO_4_. In addition, quantitative neuromuscular monitoring was not routinely available, and given the pharmacodynamic effects of magnesium on neuromuscular transmission, this may represent an additional limitation of the present study. Because magnesium administration was based on anesthesiologist preference rather than protocolized allocation, selection bias and residual confounding cannot be excluded. Pain assessments were limited to the early PACU period, and data on later postoperative pain scores, rescue analgesic requirements, and cumulative opioid consumption were not consistently available in the retrospective records. Therefore, the present study does not evaluate clinically meaningful postoperative analgesia outcomes beyond the immediate recovery phase. Because all analyzed cases were performed by a single surgeon, inter-operator variability was minimized; however, the findings may have limited generalizability to settings involving multiple operators. Finally, the in silico docking and network analyses represent static structural models that do not fully capture dynamic receptor conformations, systemic pharmacokinetics, or in vivo neurophysiological conditions; therefore, clinical extrapolation of these molecular findings should be interpreted with caution. These in silico findings are exploratory and should not be interpreted as providing mechanistic validation of the clinical observations. Accordingly, the present findings should be interpreted as hypothesis-generating, and no causal inference can be drawn. Residual or unmeasured confounding inherent to the retrospective design may also have influenced the observed findings.

## 5. Conclusions

In this retrospective cohort study, intraoperative MgSO_4_ infusion did not produce statistically significant improvements in early postoperative pain scores, intraoperative remifentanil consumption, extubation time, or time to first mobilization in patients undergoing single-level lumbar microdiscectomy. Although numerically lower pain scores and reduced opioid requirements were observed, these differences did not reach statistical significance and should be interpreted cautiously.

Complementary in silico analyses suggested structurally plausible morphine–opioid receptor interactions and stable Mg^2+^ positioning within the NMDA receptor channel pore, supporting the pharmacological rationale for magnesium-mediated modulation of nociceptive signaling. However, these exploratory in silico observations did not translate into measurable clinical benefits in this relatively low-nociceptive surgical model.

Taken together, the findings do not demonstrate a clinically meaningful benefit of intraoperative MgSO_4_ within the present cohort, despite pharmacological plausibility as an adjunctive analgesic agent.

The potential value of MgSO_4_ may be more evident in surgical settings associated with greater nociceptive burden or in patient populations at risk for enhanced central sensitization or opioid tolerance. Further prospective studies with larger sample sizes and more diverse surgical models are needed to better define the clinical role of perioperative magnesium administration within multimodal analgesia strategies.

## Figures and Tables

**Table 1:** Baseline characteristics of the study population with SMD.

Variable	Control (*n* = 19)	MgSO_4_ (*n* = 19)	*p*-Value	SMD
Age (years)	52.2 ± 14.2	47.8 ± 12.8	0.33	0.32
Sex (male/female)	10/9	10/9	1.00	0.00
BMI (kg/m^2^)	26.7 ± 5.2	28.4 ± 5.0	0.31	0.33
ASA (I/II)	13/6	13/6	1.00	0.00
Preoperative NRS	2.32 ± 0.48	2.37 ± 0.50	0.74	0.11
Symptom duration (weeks)	3.16 ± 0.96	3.00 ± 1.00	0.62	0.16
Disk level (L4–L5/L5–S1)	14/5	14/5	1.00	0.00
Surgery duration (min)	75.8 ± 12.8	77.9 ± 13.2	0.62	0.16
Comorbidities (yes/no)	6/13	5/14	1.00	0.12

SMD: standardized mean difference. Values < 0.1 indicate negligible imbalance between groups.

**Table 2:** Perioperative outcomes with mean differences and 95% confidence intervals.

Outcome	Control (Mean ± SD)	MgSO_4_ (Mean ± SD)	Cohen’s d	*p*-Value	Mean Difference	95% CI
Remifentanil consumption (µg)	319 ± 298	236 ± 166	0.34	0.270	83	−264.09 to 29.00
Extubation time (min)	4.89 ± 3.45	6.26 ± 5.03	0.31	0.320	1.37	−1.53 to 4.43
Time to first mobilization (h)	2.89 ± 0.74	2.95 ± 0.97	0.12	0.670	0.06	−0.50 to 0.70

**Table 3:** Docking interactions of morphine with μ, δ, and κ opioid receptors.

Compound	Energy Score (kcal/mol)	RMSD	H-Bond (Distance Å)	PDB ID
Morphine	−9.50	0.07	Between O-1 of TYR 328 and H-1 of morphine (1.843) Between H-1 of GLN 126 and O-1 of Morphine (1.845)	8EF6
Morphine	−8.75	0.08	Between H-1 of TYR 308 and O-1 of morphine (2.002) Between O-1 of ASP 128 and H-1 of morphine (2.105)	6PT2
Morphine	−10.38	0.06	Between H-1 of SER 323 and O-1 of morphine (2.116) Between H-1 of THR 111 and O-2 of morphine (1.990)	6VI4

**Table 4:** Topological properties of hub proteins in the extended PPI network.

Gene	Degree	Betweenness	Closeness	MCC
*GRIN2B*	10	0.17	0.96	9
*GRIA1*	9	0.12	0.95	8
*CREB1*	9	0.10	0.95	8
*BDNF*	9	0.10	0.95	8
*CAMK2A*	8	0.09	0.94	7
*GRIN1*	7	0.07	0.94	6
*GRIA2*	7	0.07	0.94	6
*AKT1*	6	0.05	0.93	5
*CACNA1A*	6	0.05	0.93	5
*MAPK1*	4	0.02	0.91	3
*CACNA1B*	4	0.02	0.91	3
*OPRM1*	3	0.01	0.90	2
*OPRD1*	1	0.00	0.85	0

**Table 5:** Selected enriched pathways identified in the PPI network.

Category	Pathway/Biological Process	Gene Count	FDR
GO Biological Process	Chemical synaptic transmission	9	8.28 × 10^−9^
GO Biological Process	Modulation of chemical synaptic transmission	8	1.63 × 10^−7^
GO Biological Process	Regulation of postsynaptic membrane potential	5	7.34 × 10^−6^
GO Biological Process	Chemical synaptic transmission, postsynaptic	5	8.42 × 10^−6^
GO Biological Process	Behavior	9	4.72 × 10^−8^
GO Molecular Function	Ionotropic glutamate receptor activity	4	8.97 × 10^−7^
GO Molecular Function	Calcium channel activity	5	4.78 × 10^−6^
KEGG Pathway	Dopaminergic synapse	8	7.76 × 10^−13^
KEGG Pathway	Long-term potentiation	6	1.67 × 10^−10^
KEGG Pathway	Nicotine addiction	6	1.13 × 10^−11^
KEGG Pathway	Amphetamine addiction	6	1.67 × 10^−10^
Reactome Pathway	Post NMDA receptor activation events	7	4.89 × 10^−11^
Reactome Pathway	CREB1 phosphorylation through NMDA signaling	5	3.48 × 10^−9^

## Data Availability

The datasets generated and/or analyzed during the current study are available from the corresponding author on reasonable request. Due to institutional regulations and ethical restrictions related to the retrospective use of patient records, individual-level clinical data cannot be made publicly available. De-identified data may be shared with qualified researchers upon reasonable request and with approval from the relevant institutional authorities. Molecular docking datasets and computational parameters used in this study are provided in the Supplementary Materials (Supplementary File S1).

## Supplementary Materials

The following supporting information can be downloaded at: https://www.mdpi.com/article/10.3390/jcm15082888/s1, Table S1: Receptor structures and docking grid parameters used for molecular docking simulations; Table S2: Multivariable linear regression analyses adjusted for age, sex, body mass index, preoperative pain score, and surgery duration; Table S3: Early PACU pain burden (AUC) comparison between groups; Table S4: Covariate balance before and after IPTW; Table S5: IPTW-weighted comparisons of clinical outcomes between groups.

## Abbreviations

The following abbreviations are used in this manuscript:

ASA	American Society of Anesthesiologists
CREB1	cAMP Response Element-Binding Protein 1
FDR	False Discovery Rate
GO	Gene Ontology
KEGG	Kyoto Encyclopedia of Genes and Genomes
MCC	Maximal Clique Centrality
NMDA	N-Methyl-D-Aspartate
NRS	Numeric Rating Scale
PACU	Post-Anesthesia Care Unit
PPI	Protein–Protein Interaction
