# Supplementary material for: Intraoperative Magnesium Sulfate and Early Postoperative Analgesia in Lumbar Microdiscectomy: A Retrospective Clinical Study Integrating Molecular Docking and Protein Interaction Network Analysis
Source: J Clin Med. 2026 Apr 10;15(8):2888. doi: 10.3390/jcm15082888 (PMC13116607; doi:10.3390/jcm15082888)
Supplement: Supplementary file 1 [file jcm-15-02888-s001.zip › jcm-4228487-supplementary.pdf]

Supplementary Materials

Intraoperative Magnesium Sulfate and Early Postoperative Analgesia in Lumbar Microdiscectomy: A Retrospective Clinical Study Integrating Molecular Docking and Protein Interaction Network Analysis

Supplementary Methods: Molecular Docking Analysis

S1. Ligand Preparation

The chemical structure of morphine was constructed using ChemDraw Ultra 12.0. The ligand geometry was optimized by energy minimization using the MM2 force field implemented in ChemBio Ultra 13.0 in order to obtain a stable conformational state. The optimized ligand structure was exported in PDB format and subsequently converted to PDBQT format using Open Babel v3.1.1, which is required for AutoDock-based docking simulations.

S2. Docking Protocol Validation

To validate the docking protocol, redocking of the co-crystallized ligand was performed for each receptor structure used in this study. The crystallographic ligand was first removed from the receptor structure, and docking was subsequently carried out using the same grid parameters defined for the morphine docking simulations.

The root mean square deviation (RMSD) between the docked ligand pose and the experimentally determined crystallographic pose was calculated.

An RMSD value below 2.0 Å was considered indicative of reliable docking performance and appropriate reproduction of the experimentally observed binding orientation.

RMSD values represent the deviation of docked poses from the lowest-energy conformation within each docking cluster.

S3. Molecular Docking with the μ-Opioid Receptor (MOR)

The three-dimensional crystal structure of the μ-opioid receptor (MOR) was retrieved from the Protein Data Bank (PDB ID: 8EF6) and used as the target protein for morphine docking.

The receptor structure contained chains A, B, C, E, F, M, and R and was crystallized in complex with an agonist ligand.

Protein preparation involved the following steps:

• removal of all crystallographic water molecules

• addition of polar hydrogen atoms

• assignment of Gasteiger partial charges

Docking simulations were performed using AutoDock version 4.2.6.

The docking search space was defined using a cubic grid box centered on the experimentally identified ligand-binding pocket corresponding to the position of the co-crystallized ligand.

Grid center coordinates:

x = 138.916

y = 124.732

z = 133.938 Å

Grid box size:

40 × 40 × 40 Å

Grid spacing:

0.375 Å

S4. Molecular Docking with the δ-Opioid Receptor (DOR)

The crystal structure of the δ-opioid receptor (DOR) was obtained from the Protein Data Bank (PDB ID: 6PT2).

The receptor structure contained chains A, B, C, and D and was crystallized with an agonist ligand.

Protein preparation followed the same procedure used for MOR:

• removal of crystallographic water molecules

• addition of polar hydrogens

• assignment of Gasteiger charges

Grid center coordinates:

x = −19.806

y = 41.639

z = −27.722 Å

Grid box size:

40 × 40 × 40 Å

Grid spacing:

0.375 Å

Docking simulations were performed within the ligand-binding region defined by the crystallographic ligand.

S5. Molecular Docking with the κ-Opioid Receptor (KOR)

The crystal structure of the κ-opioid receptor (KOR) was retrieved from the Protein Data Bank (PDB ID: 6VI4).

The receptor contained chains A, B, C, and D and was crystallized in complex with an agonist ligand.

Protein preparation included:

- removal of crystallographic water molecules
- addition of polar hydrogens
- assignment of Gasteiger partial charges

Grid center coordinates:

x = 29.556

y = −63.139

z = −21.111 Å

Grid box size:

40 × 40 × 40 Å

Grid spacing:

0.375 Å

Docking simulations were performed within the ligand-binding pocket identified from the crystallographic ligand.

S6. Docking Parameters and Search Algorithm

Docking simulations were performed using the Lamarckian Genetic Algorithm (LGA) implemented in AutoDock version 4.2.6.

The following search parameters were applied:

- Number of independent docking runs: 20.
- Genetic algorithm search method: Lamarckian Genetic Algorithm
- Population size and energy evaluation parameters: default AutoDock settings

Each ligand–receptor complex was explored through 20 independent docking simulations, generating multiple conformational poses within the defined binding pocket.

All receptor structures were selected based on high crystallographic resolution and the availability of co-crystallized ligands defining the orthosteric binding pocket used for grid placement. Together with the redocking validation and standardized grid parameters applied across receptors, these procedures were implemented to ensure methodological consistency and reproducibility of the docking simulations, while acknowledging that docking results provide mechanistic hypotheses rather than definitive predictions of in vivo pharmacological interactions.

The number of docking runs was selected to ensure adequate conformational sampling within the defined binding pocket.

S7. Structural Analysis of the NMDA Receptor Channel

In addition to opioid receptor docking, structural inspection of the human GluN1/GluN2B NMDA receptor channel pore (PDB ID: 9IYP) was performed to evaluate potential Mg^{2+} coordination sites within the ion channel region.

No ligand docking simulations were performed for the NMDA receptor. The NMDA receptor structure was analyzed only for qualitative structural interpretation and was not included in the docking calculations.

This analysis focused on the structural arrangement of residues lining the channel pore that contribute to the voltage-dependent Mg^{2+} blockade characteristic of NMDA receptor physiology.

S8. Software and Computational Tools

Ligand preparation and docking simulations were performed using the following software tools:

- ChemDraw Ultra 12.0 – ligand structure construction
- ChemBio Ultra 13.0 – MM2 energy minimization
- Open Babel v3.1.1 – file format conversion (PDB → PDBQT)

• AutoDock version 4.2.6 – molecular docking simulations

These software tools were applied sequentially for ligand construction, receptor preprocessing, docking simulations, and structural interaction analysis.

Supplementary Tables

Table S1. Receptor structures and docking grid parameters used for molecular docking simulations.

Receptor	PDB ID	Chains	Grid center (x, y, z)	Grid box size (Å)	Grid spacing (Å)
μ-Opioid receptor (MOR)	8EF6	A, B, C, E, F, M, R	138.916, 124.732, 133.938	40 × 40 × 40	0.375
δ-Opioid receptor (DOR)	6PT2	A, B, C, D	−19.806, 41.639, −27.722	40 × 40 × 40	0.375
κ-Opioid receptor (KOR)	6VI4	A, B, C, D	29.556, −63.139, −21.111	40 × 40 × 40	0.375

The docking parameters listed in this table correspond to the grid definitions used for receptor–ligand docking during the molecular docking simulations.

S9. Supplementary Statistical Analyses

To further address potential confounding related to the retrospective study design, additional exploratory statistical analyses were performed. These analyses were considered supplementary and not intended for causal inference.

Note. Postoperative pain scores reported in the main manuscript correspond to measurements obtained at PACU arrival (0 min) and at 5, 10, 15, and 30 minutes. This clarification ensures consistency between the Methods, Results, and Figure 2.

Propensity score–based analyses were performed as exploratory supplementary analyses. Propensity scores were estimated using logistic regression including age, sex, body mass index, preoperative pain score, and surgery duration as covariates. Inverse probability of treatment weighting (IPTW) was then applied to create a weighted pseudo-population.

Table S2. Multivariable linear regression analyses adjusted for age, sex, body mass index, preoperative pain score, and surgery duration.

Outcome	Adjusted β	95% CI	p-value
PACU pain AUC	−11.86	−34.94 to 11.22	0.303
Remifentanil consumption	−117.55	−264.09 to 29.00	0.112
Extubation time	1.45	−1.53 to 4.43	0.329
Time to first mobilization	0.10	−0.50 to 0.70	0.737

Table S3. Early PACU pain burden (AUC) comparison between groups.

Group	Mean ± SD
Control	97.2 ± 32.1
$MgSO_4$	85.3 ± 28.4

Mean difference: −11.86
95% CI: −34.94 to 11.22
p = 0.303

Table S4. Covariate balance before and after IPTW.

Variable	SMD before	SMD after IPTW
Age	0.27	0.06
Sex	0.00	0.01
BMI	0.34	0.01

SMD: standardized mean difference. Values <0.1 indicate adequate balance between groups.

Table S5. IPTW-weighted comparisons of clinical outcomes between groups.

Outcome	β	95% CI	p-value
PACU AUC	−11.68	−31.14 to 7.78	0.240
Remifentanil	−137.72	−409.77 to 134.34	0.321
Extubation time	1.39	−1.30 to 4.08	0.311
Time to first mobilization	0.09	−0.54 to 0.73	0.777

These analyses were exploratory and limited by the small sample size and restricted set of available covariates; therefore, results should be interpreted cautiously and not considered causal.
